# Collie Eye Anomaly in Australian Kelpie dogs in Poland

**DOI:** 10.1186/s12917-019-2143-y

**Published:** 2019-11-04

**Authors:** Natalia Kucharczyk, Anna Cislo-Pakuluk, Peter Bedford

**Affiliations:** 1 Viva Veterinary Clinic, Wroclaw, Poland; 2 Royal Veterinary College, London, UK

**Keywords:** Australian Kelpie, Choroidal hypoplasia, Collie Eye Anomaly, *NHEJ1* gene

## Abstract

**Background:**

To report the occurrence of choroidal hypoplasia in the Australian Kelpie breed in Poland, the affected dogs testing positive for the Collie Eye Anomaly *NHEJ1* gene mutation.

**Case presentations:**

Choroidal hypoplasia (CH) was initially diagnosed in a young female Australian Kelpie presented for routine ophthalmological examination prior to breeding. Indirect ophthalmoscopy revealed tigroid fundi bilaterally with areas of abnormally arranged choroidal vasculature temporal to the optic disc. These lesions had the appearance of the choroidal hypoplasia diagnostic for Collie Eye Anomaly, a genetically determined disease seen most commonly in Collie types.

The DNA based test for the *NHEJ1* gene mutation that is confirmatory for Collie Eye Anomaly proved the dog to be homozygous for this mutation. Twenty one other related dogs were subsequently examined genetically, the dam proving to be affected and eight others were shown to be carriers.

**Conclusions:**

This report demonstrates that Collie Eye Anomaly is present in a Polish bred Australian Kelpie line and as such breeders in this country and those importing dogs or semen internationally should be aware of other possible cases.

## Background

Collie Eye Anomaly (CEA) is a congenital canine pleomorphic ocular disease characterized by two main lesions, choroidal hypoplasia/chorioretinal dysplasia (CH/CRD) and papillary/peripapillary colobomata. CH/CRD, referred to as CH in this paper, is characterized by the focal absence of pigmented choroidal tissue and tapetum temporal to the optic disc and the presence of choroidal blood vessels abnormal in both appearance and arrangement. If the lesion also involves part of the non-tapetal fundus the overlying retinal pigment epithelium lacks pigment. CH is always bilaterally present, but to varying degrees between affected dogs and even within the same individual. However, no matter how extensive, CH appears to be of no clinical significance in terms of an effect on sight. Colobomatous defects can vary considerably in size, the larger lesions affecting vision and potentially being involved in post-natal retinal detachment. In addition to these two features both congenital and post-natal retinal detachment and intraocular haemorrhage are also described, but although potentially blinding, these features are of low incidence [1–5]. Thus CEA affected dogs can vary from mildly to moderately affected without vision impairment or possibly present with partial or total blindness.

A number of studies concerning the genetic background of the disease have been completed [6–12], one initial suggestion being that CEA might be inherited as a complex trait involving multiple genetic factors [5]. Subsequently it has been shown to be an autosomal recessive trait in Collie types and Lowe et al. localized a 3.9 - cM locus associated with CH on chromosome 37 [9]. Fine - mapping techniques have been used to identify a 7.8 kb deletion in intron 4 of the *NHEJ1* gene(non-homologous end-joining factor 1), the CEA locus [10]. CH has been shown to be due to the same *NHEJ1* deletion in several other breeds and a confirmatory genetic test is now commercially available.

CEA was first described as an hereditary disorder in the Border Collie, Rough and Smooth Collies and the Shetland Sheepdog [1–5, 13, 14]. It has also been variously reported in several other breeds including the Australian Shepherd, the Boykin Spaniel, the Lancashire Heeler, the Longhaired Whippet, the Nova Scotia Duck Tolling Retriever, the Hokkaido dog and the Silken Windhound [15–20]. A phenotypically identical lesion has been seen in other non-collie breeds including the German Shepherd Dog, Miniature and Toy Poodles, the Beagle and a mixed-breed dog [17, 18, 20]. The causal genetic defect has not been determined for all of these breeds.

This is the first published report of CEA in the Australian Kelpie breed. The initial ophthalmoscopic finding was subsequently confirmed using the specific DNA based test. Based on analysis of the probands’ pedigree the dog’s dam proved to be homozygous for the mutation and one other affected dog plus several carriers were found.

## Case presentation

**Fig. 1:**
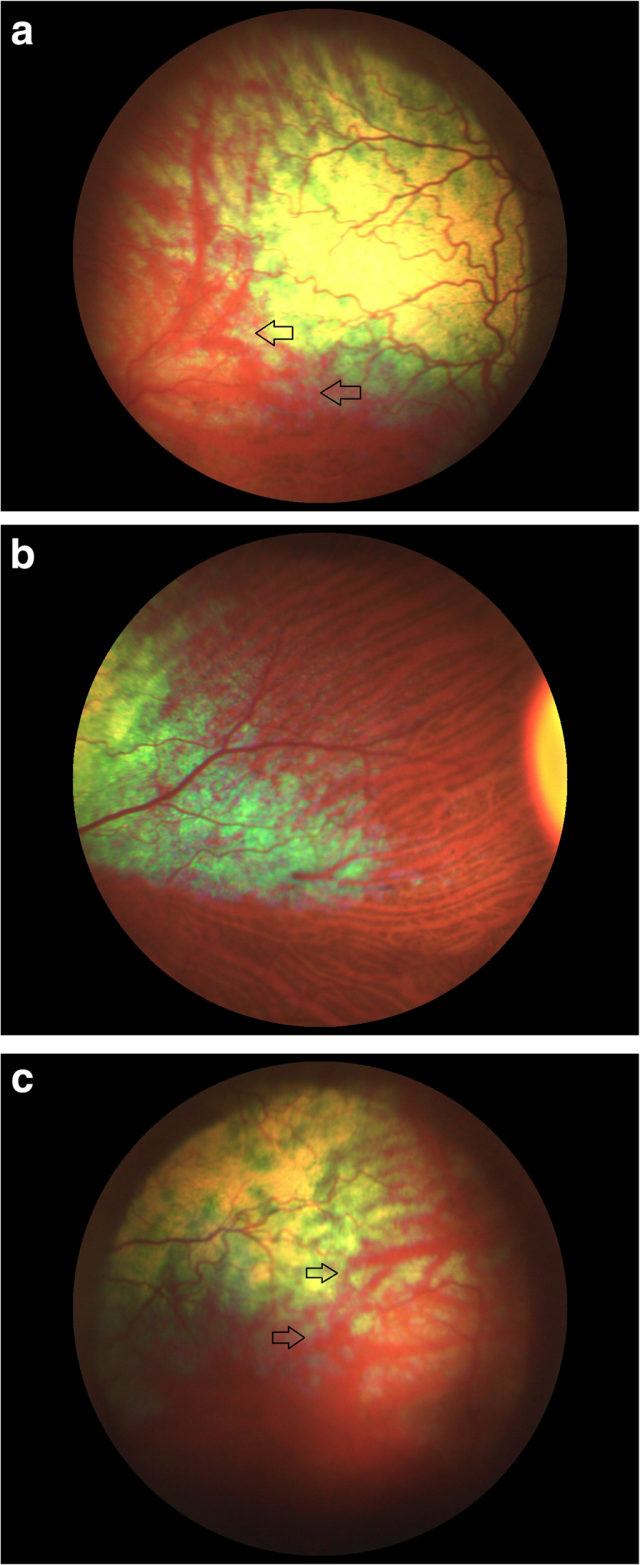
Fundus photographs of the affected female to demonstrate the abnormal arrangement of choroidal vessels in the temporal tapetal fundus. **a** Left eye, temporal area, the arrowsdemonstrating the large area of choroidal hypoplasia. **b** Right eye, medial area, demonstrating a normal appearance. **c** Left eye, temporal area, demonstrating choroidal hypoplasia

The initial finding was in a two-and-a-half year old female Australian Kelpie which was being considered for breeding purposes. There were no reported visual deficits and performance in a maze test, the menace response, the pupillary light reflexes and the dazzle reflex were all considered to be normal. Biomicroscopic examination (SL-17 Portable Slit Lamp, Kowa) of the anterior segment revealed minor bilateral stromal iridal atrophy. Mydriasis was effected using tropicamide (Tropicamidum WZF 1%, Polfa Warszawa) and fundus examination completed using direct and indirect ophthalmoscopy (Keeler Standard Direct and Keeler Vantage Plus Indirect ophthalmoscopes). Fundus imaging was obtained using a 30D condensing lens (Volk 2) and photographs were taken using the ClearView Fundus Camera (Optibrand). Both fundi had a tigroid appearance in the non-tapetal areas, but an irregular arrangement of the choroidal vasculature was observed bilaterally in the temporal region of the tapetal fundus. The lesions were easily identifiable with the choroidal blood vessels being fewer in number and thicker than normal. The degree of CH allowed the white appearance of the sclera to be visible between the abnormal blood vessels (Fig. 1a-c). Colobomatous lesions were not present and no other ocular abnormalities were found. Based on the presence of CH a tentative diagnosis of CEA was suggested, this diagnosis being subsequently confirmed using a real time PCR DNA test for the CEA *NHEJ1* 7.8 kb deletion gene mutation (Laboklin GmbH & Co.KG, 8304).

**Fig. 2:**
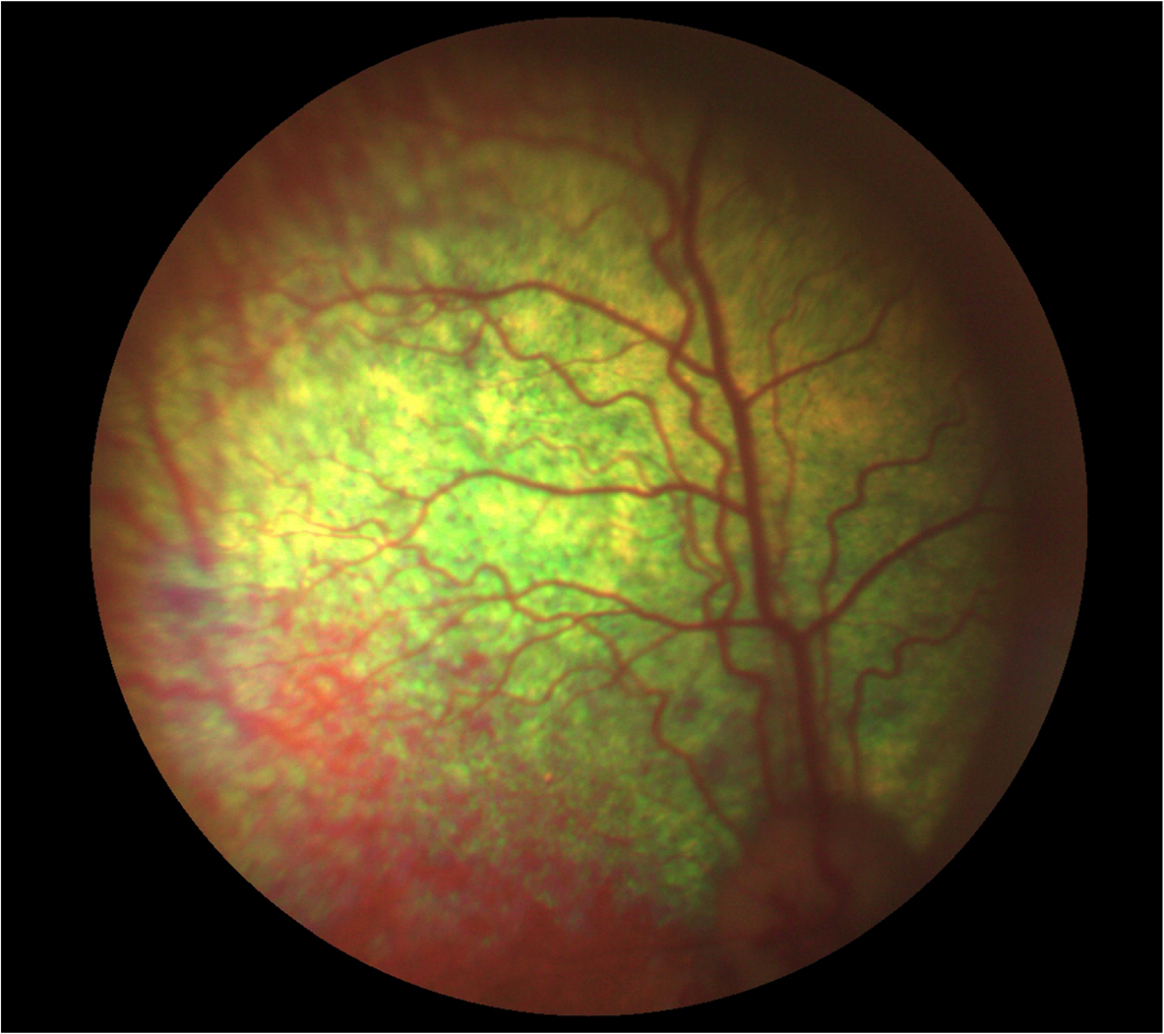
The temporal area of the right fundus of the affected dam demonstrating a small area of choroidal hypoplasia

**Fig. 3:**
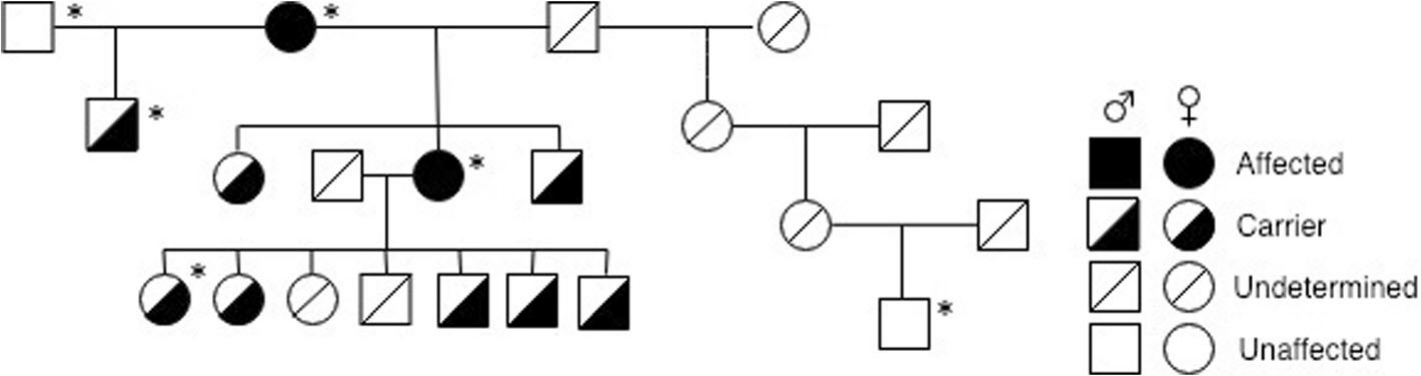
Pedigree analysis of the 21 related Australian Kelpie dogs examined on the survey. The subject of this case report is the affected dog on the second line and asterisks mark the six dogs clinically examined

Using an analysis of the probands’ pedigree twenty other dogs related to the affected female were subsequently screened using the DNA test for the CEA mutation. Five of these dogs were also examined clinically and CH lesions were found in one, the dog’s dam. Here the small size of lesions rendered a positive ophthalmoscopic diagnosis difficult (Fig. 2), demonstrating the value of litter screening at six to 8 weeks of age to rule out a putative “go normal” diagnosis. However DNA testing confirmed the CEA diagnosis for this dog whilst eight others were shown to be carriers for the disease (Fig. 3). Colobomatous lesions and other possible features of CEA were not found in any of these dogs.

Permission has been obtained from all the owners and breeders whose dogs were involved in this study to record and publish both the clinical findings and the DNA results.

## Discussion and conclusions

This is the first report of CEA in the Australian Kelpie breed, diagnosed on the basis of bilateral CH seen ophthalmoscopically and confirmed by genetic testing. The breed originated from British dogs imported into Australia for stock work in the early nineteenth century. They were simply described as collies or colleys, before the more traditional “collie” breeds were established. This background indicated that CEA could be present in the Kelpie and was the reason for this small study.CH lesions and the association with the CEA related *NHEJ1* mutation could be reported,but colobomatous defects and the other features of CEA were not seen in either of the affected dogs. Further screening is required to evaluate the prevalence of the *NEHJ1* gene mutation in this breed and breeders should be aware that in addition to the use of the DNA test and the ophthalmoscopic examination of the breeding stock, litter screening at six to 8 weeks of age will identify both CH and possible colobomatous lesions. The existence of a “go normal” change in which CH lesions can be masked by pigmentation beyond 12 weeks of age means that in adult dogs ophthalmoscopic examination needs to be supported by DNA testing to effect efficient disease control.

## Data Availability

The datasets used during the current study are available from the corresponding author on reasonable request.

## Abbreviations

CEA: Collie Eye Anomaly
CH: Choroidal hypoplasia, focal absence of choroidal tissue temporal to the optic disc
CRD: Chorioretinal dysplasia
DNA: Deoxyribonucleic acid
*NHEJ1*: Non-homologous end-joining factor 1
